# Marginal Bone Loss Around the Implant: A Retrospective Analysis of Bone Remodeling Over Five Years of Follow-Up

**DOI:** 10.7759/cureus.76228

**Published:** 2024-12-22

**Authors:** Cosmin I Faur, Adrian Herman, Ionut Leahu, Sergiu Megiesan, Ionut Caluian

**Affiliations:** 1 Regina Maria Dental Department, Regina Maria Private Healthcare Network, Bucharest, ROU; 2 Faculty of Medicine and Pharmacy, University of Oradea, Oradea, ROU; 3 Mathematics Department, Imperial College London Alumni, London, GBR

**Keywords:** bone remodeling, bone resorption, dental implant failure, marginal bone loss, peri-implantitis

## Abstract

Introduction: Bone remodeling around implants in implant-supported rehabilitation is a continuous debate with no consensus in the literature. This study aimed to investigate the implant- and patient-specific factors contributing to marginal bone loss near the implant.

Materials and methods: We included patients who had implant-supported prosthetic rehabilitation using one implant system, between 2014 and 2018, who had full follow-up documentation and orthopantomography over five years, and who had no unwell-controlled systemic pathologies that may influence bone metabolism.

Results: Eighty-one patients who received 500 implants met the inclusion criteria. We observed approximately 1 mm of bone resorption at the five-year follow-up, with the first 0.78 mm of them being documented at the three-year follow-up. Adults younger than 60 years old had an increase in bone resorption by approximately 30%. No difference was seen between men and women. However, a slight increase in bone resorption at five years was seen in female patients older than 50 years old than in ones younger than 50 years (by 30%). The narrowest diameter (3.5 mm; p = 0.001) and anterior mandible (p = 0.008) had the highest bone resorptions. Contrarily, with an insertion depth of approximately 1 mm (p = 0.004), the splinted implant prosthesis (p = 0.21) and zirconia material of the prosthesis (p = 0.57) had the lowest bone remodeling. Moreover, patients younger than 60 years and female patients above 50 years had an increased bone resorption.

Conclusions: Bone remodeling is a multifactorial process. The treatment planning has to take into consideration both implant- and patient-specific factors.

## Introduction

Implant-prosthetic restoration of edentate spaces is nowadays the golden standard treatment for oral rehabilitation due to its high success rates, physiologic mimetics, and increased quality of life [1,2]. The osteointegration, a controversial concept that is responsible for the favorable outcomes of the treatment, entails the inherent bone reorganization and remodeling around the inserted implant [3-5]. A 1 mm bone resorption around the implant in the first year and 0.2 mm furtherly are accepted as criteria for a successful treatment [2,6]. However, many factors are involved in marginal bone remodeling.

The factors that influence marginal bone loss are divided into systemic and local. On the one side, systemic diseases, such as diabetes mellitus or immunosuppression, can be absolute or temporary contraindications of implant-prosthetic oral rehabilitation. The metabolic and vascularization changes induced by systemic pathologies interfere with bone healing and may increase the risk of local infection [7]. All systemic diseases have to be well-controlled at the moment of implant insertion; otherwise, the implant treatment may fail. Also, systemic effects of various habits, such as cigarette smoking, may interfere with local vascularization and the healing process and, hence, increase the risk or implant failure [8,9].

On the other side, local factors that influence bone resorption are related to oral diseases, such as periodontitis, or they can be related to implants, such as the anatomical location (e.g., the maxilla or mandible), length or diameter of the implant, 3D implant positioning, depth of the implant insertion, or type of implant (e.g., subcrestal, bone level, or tissue level) [10]. Some authors indicated that factors, such as thin gingiva, deep insertion, or short abutments, increase bone remodeling in the early post-implantation period in subcrestal implants [11]. Also, the density of bone resorption around the implant in the early time can be a prognostic factor for implant failure [12]. However, the results of the literature are controversial [7]. While some authors indicated the optimal depth position of the implant at 1-2 mm subcrestal, others consider that deeper implant insertion is prone to greater bone remodeling and more intense local inflammatory status [13,14]. Moreover, prosthetic materials may influence bone remodeling around the implant. For example, zirconia may increase bone resorption around the implant. The remodeling depends not exclusively on the type of material but also on the prosthetic design.

However, bone remodeling is inherent, and high efforts are made to understand and prevent bone resorption near the implant and the associated peri-implantitis. The heterogeneous literature results are due to the different approaches to marginal bone loss evaluation, various implant systems, or different protocols for implant insertion [15-24].

The aim of the present study was to evaluate and to quantify the patient- and implant-specific factors that contribute to marginal bone remodeling around the implant using one implant system.

## Materials and methods

Patients who presented in the Dental Offices of Regina Maria Dental Department (Romania) for prosthetic-implant oral rehabilitation between January 2014 and December 2018 were proposed for this study. The inclusion criteria of this retrospective investigation are presented in Table 1 [7]. Only patients who met all the inclusion criteria and received Bredent Implants (Blue Sky, Narrow Sky, or Classic Sky, Bredent Medical GmbH & Co., Senden, Germany) were included in this study [25]. We excluded patients with unwell-controlled systemic pathologies, such as lipid metabolism changes (e.g., high level of low-density lipoprotein (LDL)), diabetes mellitus (e.g., glycated hemoglobin (HbA1c) >7 mg/dl), or other pathologies that may interfere with the implant treatment and bone metabolism (e.g., immunosuppression) [11,26]. Also, patients with cemented prosthetics or incomplete data were excluded. All the patients were prepared for implant placement using cone-beam computed tomography (CBCT) and were further evaluated by orthopantomography (OPG, Planmeca, Helsinki, Finland) at T0 (immediately after the surgery), T1 (three-year follow-up), and T2 (five-year follow-up). The bone measurements were performed for each period as described below in "Bone remodeling measurements". The patients' demographic data (e.g., age, sex), including cigarette smoking habits, were obtained from the informatic system. The patients were classified as smokers and non-smokers, with the smokers being also named as light (<5 smoking index, cigarettes per day × years of smoking), medium (5-20 smoking index), and heavy (>20 smoking index).

**Table 1:** Inclusion criteria OPG: orthopantomography; T0: immediately after the surgery, T1: three-year follow-up; T2: five-year follow-up

Criteria for inclusion	
1	Minimum of one implant placement with or without bone augmentation
2	Available medical records
3	Available radiological records (OPG) at the three moments of evaluation (T0, T1, and T2)
4	Available clinical data of implant-prosthetic restoration
5	Screw-retained prosthetic restoration
6	Minimum five years of follow-up
7	Patients aged above 18 years old in general good health

This study was approved by the Ethics Committee of Regina Maria Private Healthcare Network (approval number: 617/28.10.2024), in accord with the updated Declaration of Helsinki.

Implant insertion protocol

All the patients signed the informed consent before the procedure. Three experienced oral surgeons (with a minimum of three years of implantology experience and a minimum of 1000 implants applied) performed the implant insertions following the same protocol. The anxious patients received conscious sedation during the surgery. The mouth was rinsed with an antimicrobial solution of 0.2% chlorhexidine (Corsodyl; Glaxo Smith Kline, Zeist, the Netherlands). Local anesthetic infiltration was administrated (Ubistesin; 3M Deutschland GmbH, Neuss, Germany) using a periapical technique. Then, the surgeon performed a crestal incision with minimal releasing mesial and/or distal incisions in case of multiple implant insertions. The protocol for implant drilling and implant insertion was in accordance with the manufacturer's indications (Bredent protocol) [15]. All the implants were inserted at 0.5-2 mm subcrestally with a torque of 35-70 Ncm. In the sinus lift augmentation, as well as buccal augmentation to increase the bone width, bone grafting material (Bio‐Oss; Geistlich Pharma AG, Wolhusen, Switzerland) was used in association with plasma rich in growth factors (PRGF) Endoret (BTI Biotechnology Institute, Vitoria-Gasteiz, Spain) and collagen resorbable membrane (Evolution, OsteoBiol, Torino, Italy). Flaps were sutured using a combination of absorbable and non-absorbable (BIOPRO and PDO'x, Biosintex, Ilfov, Romania) threads in an interrupted fashion suture. In the case of the all-on X implant technique insertion, the implants were loaded with provisional restorations (polymethyl methacrylate (PMMA)) using multi-unit abutment (MUA), except for the implants that were inserted in addition to a sinus lift augmentation. The rest of the implants received cover screws or gingiva healing screws. Antibiotic (amoxicillin 1 g 2 × 1 dose/day or clindamycin 600 mg 2 × 1 dose/day) and antalgic (ketoprofen 100 mg 2 × 1 dose/day) treatments were administered for seven days. The implants with cover screw had a second surgery at six months for applying healing abutments. After rinsing the mouth with an antiseptic solution and local periapical anesthesia, a crestal incision was performed, and the cover screw was replaced with the healing abutment; interrupted sutures were applied. After two weeks of mucosa healing, the restoration protocol had started. The materials used for restorations were as follows: full-contour zirconia, metal-ceramic, Emax (Baluke Dental Laboratory, Richmond Hill, Canada), and BioHPP (Bredent UK Ltd, Chesterfield, UK). All the restorations were screw-retained. Follow-up protocol and maintenance program were set.

Bone remodeling measurements

**Figure 1:**
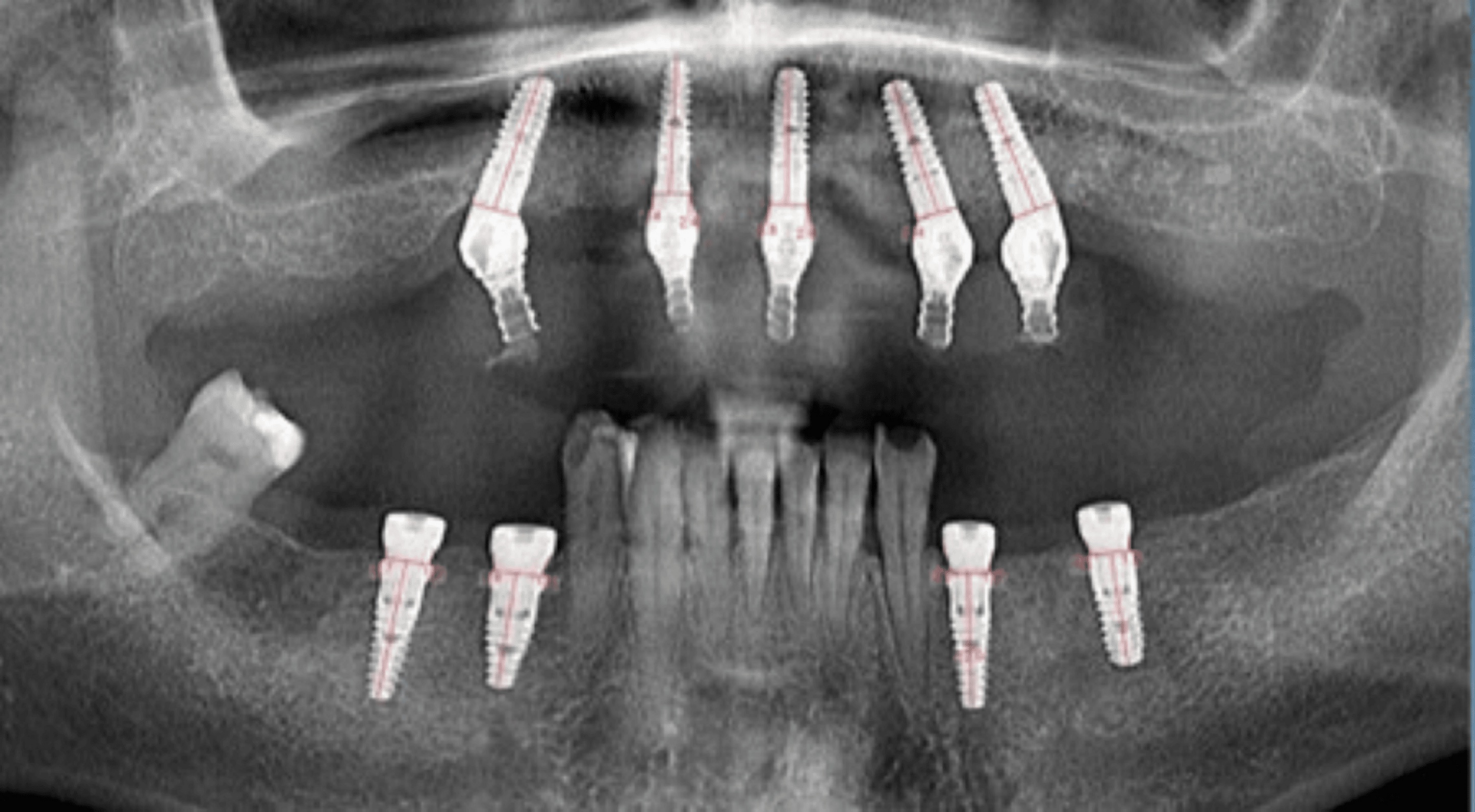
Vertical measurements (red lines), five-year follow-up

**Figure 2:**
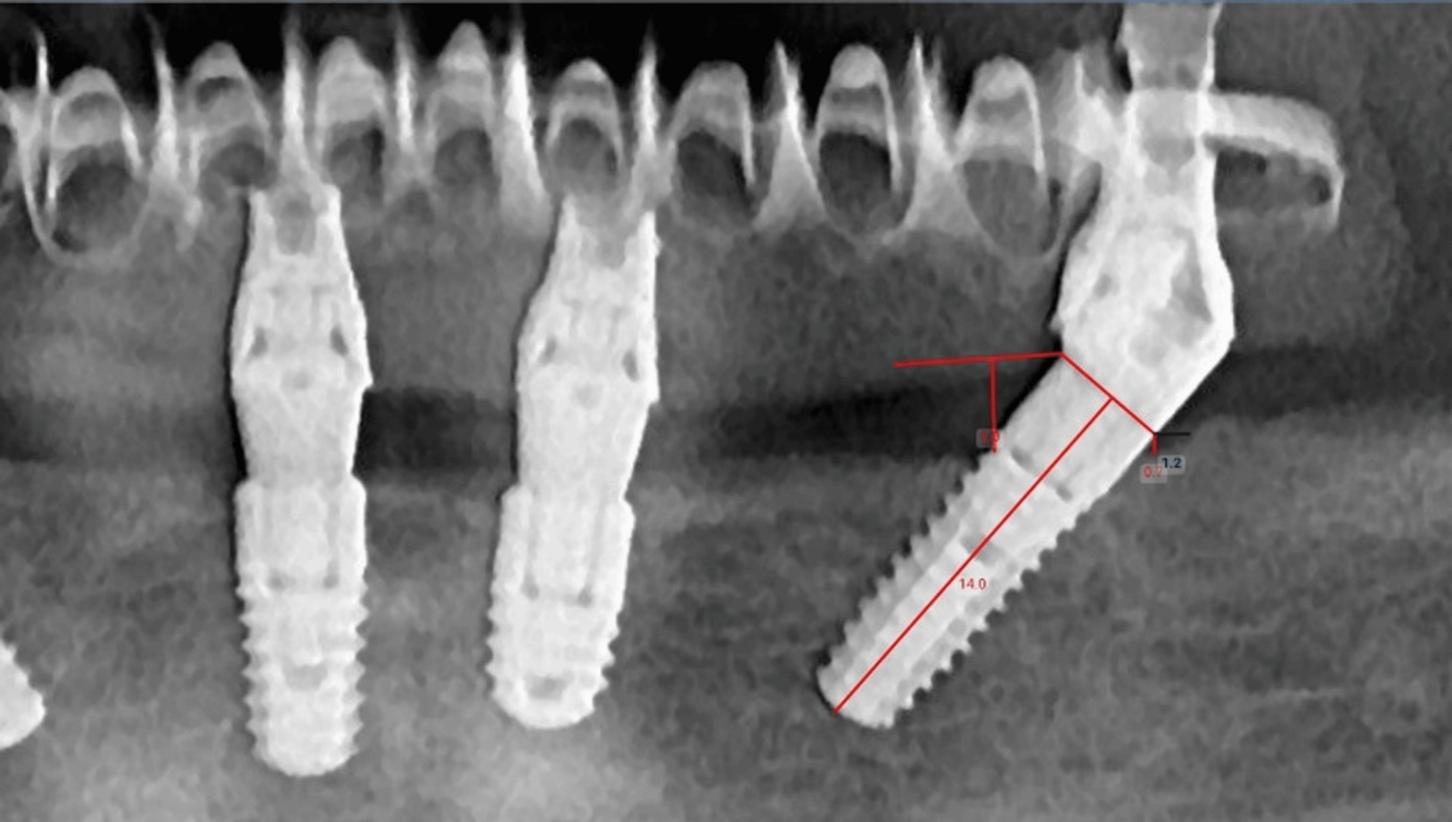
Horizontal measurements (blue line, distally at the position of 35 angulated implant), five-year follow-up

The measurements were digitally performed on the OPG by the same evaluator (I.C.) using a digital ruler [27]. A horizontal line was drawn at the implant platform, and a vertical measurement on the mesial and distal aspect of the implant was performed between the platform line and the adjacent crestal bone (Figure 1). Also, a vertical line was set at the mesial and distal aspects of the implant, and the measurements were done between these lines and adjacent bone (Figure 2). A mean between mesial and distal measurements was calculated for vertical and horizontal measurements. The bone remodeling value was then calculated for each measurement using the formulas T0-T1 for the bone remodeling after three years and T0-T2 for five years.

Statistics

The statistical analysis was performed using Python 3.10 (Jupyter Notebook, New York, USA) software and the following techniques: statistical tests (one-way ANOVA, independent t-test), Pearson's correlation coefficient, trend visualization (linear regression), and histograms. A p-value of <0.05 was set as the statistical significance threshold.

## Results

A total of 81 patients who had 500 Bredent implants (442 Blue Sky, 51 Classic Sky, and 7 Narrow Sky) were included in this study (Table 2). Most patients were female (51 subjects) with a male-to-female ratio of 1.7:1. No variation in bone remodeling was observed between men and women (p > 0.05). The patients' ages ranged from 39 to 79 years old, with a mean and standard deviation of 57 ± 9 years. Patients received between one and 12 implants, with a mean of approximately six implants per patient. Patients older than 60 years had more implants per patient than those younger than 60 years old (5.9 implants per patient vs 6.5 implants per patient). Twenty-five out of 35 patients above 60 years old had an All-on-4 or All-on-6 implant concept compared with 17 out of 46 patients younger than 60 years old. Thirty-two out of 42 patients had bimaxillary implants for total-implant rehabilitation in one surgical session under sedation. In the mandible, most patients received four implants for total jaw implant rehabilitation (39 patients with four implants and three patients with six implants) compared with the maxilla where six implants were required (32 patients with six implants).

**Table 2:** Demographic and implant features of the cohorts Observations: * one patient could have different types of implants; ** mean ± standard deviation of group values at different time intervals. Legend: p-value and F/t the test's statistic for ANOVA and t-test, respectively. mm: millimeters; T0: immediately after the surgery, T1: three-year follow-up; T2: five-year follow-up

	Patients (n=%)*	Implants (n=%)	Vertical (mm)						Horizontal (mm)					
			T0-T1 bone resorption**	P	F/t	T0-T2 bone resorption**	P	F/t	T0-T1 bone resorption**	P	F/t	T0-T2 bone resorption**	P	F/t
Total implants	81	500	0.78 ± 0.99	-		1.03 ± 1.09	-		0.16 ± 0.58	-		0.19 ± 0.63	-	
Age														
Mature adults (<60 years old)	45	266	0.87 ± 0.99	0.02	5.22	1.06 ± 1.07	0.48	0.47	0.2 ± 0.66	0.09	2.82	0.21 ± 0.66	0.47	0.51
Elderly (>61 years old)	36	234	0.67 ± 0.98			0.99 ± 1.1			0.11 ± 0.47			0.17 ± 0.59		
Sex														
Female	51	325	0.76 ± 1.03	0.81	0.05	0.76 ± 1.11	0.53	0.38	0.17 ± 0.62	0.76	0.08	0.19 ± 0.63	0.73	0.11
Male	30	175	0.79 ± 0.92			0.99 ± 1.04			0.15 ± 0.51			0.21 ± 0.62		
Cigarette smoking														
No	63	381	0.79 ± 1	0.73	0.11	1.05 ± 1.11	0.51	0.51	0.17 ± 0.64	0.46	0.52	0.21 ± 0.69	0.28	1.16
Yes	18	119	0.75 ± 0.97			0.97 ± 1.03			0.13 ± 0.35			0.14 ± 0.41		
Type of implant														
Blue Sky	77	442	0.76 ± 0.98	0.43	0.82	1.04 ± 1.09	0.83	0.17	0.11 ± 0.42	<0.001	14.49	0.16 ± 0.63	0.08	4.79
Classic Sky	19	51	0.90 ± 1.05			0.95 ± 1.09			0.56 ± 1.24			0.45 ± 1.18		
Narrow Sky	4	7	1.1 ± 1.12			1.15 ± 1.51			0.29 ± 0.37			0.32 ± 0.41		
Diameter (mm)														
3.5	32	78	1.17 ± 1.22	0.008	7.15	1.47 ± 1.28	0.003	8.01	0.26 ± 0.85	0.24	1.39	0.21 ± 0.72	0.75	0.27
4	79	400	0.71 ± 0.94			0.94 ± 1.04			0.14 ± 0.52			0.18 ± 61		
4.5	18	22	0.68 ± 066			1.13 ± 0.9			0.12 ± 0.43			0.28 ± 0.67		
Length (mm)														
8	17	35	0.55 ± 0.72	0.08	2.19	0.65 ± 0.82	0.14	1.80	0.11 ± 0.57	0.1	2.05	0.18 ± 0.67	0.007	4.08
10	57	139	0.65 ± 0.99			1 ± 1.12			0.24 ± 0.66			0.34 ± 0.79		
12	60	208	0.83 ± 0.94			1.07 ± 1.07			0.17 ± 0.63			0.17 ± 0.57		
14	35	118	0.90 ± 1.13			1.11 ± 1.14			0.07 ± 0.34			0.06 ± 0.45		
Depth of implant insertion regarding the crestal bone														
<1 mm	76	331	0.68 ± 0.9	0.004	5.49	0.91 ± 1.05	0.003	7.98	0.18 ± 0.66	0.52	0.64	0.21 ± 0.71	0.23	1.43
1-2 mm	60	121	1.02 ± 1.12			1.37 ± 1.06			0.14 ± 0.39			0.20 ± 0.49		
2 mm	25	48	0.87 ± 1.15			0.99 ± 1.26			0.08 ± 0.3			0.05 ± 0.19		
Position of the implant														
Mandible	80	223	0.86 ± 1.07	0.09	1.61	1.10 ± 1.18	0.23	1.20	0.23 ± 0.81	0.02	2.31	0.31 ± 0.87	< 0.001	3.46
Maxilla	78	277	0.71 ± 0.92			0.98 ± 1.01			0.10 ± 0.28			0.10 ± 0.31		
Posterior mandible	62	156	0.70 ± 0.95	0.001	5.50	0.92 ± 1.09	0.002	6.66	0.33 ± 0.95	<0.001	7.75	0.43 ± 1.01	<0.001	11.28
Posterior maxilla	52	174	0.71 ± 0.94			0.87 ± 0.95			0.14 ± 0.33			0.13 ± 0.37		
Anterior mandible	34	67	1.23 ± 1.21			1.51 ± 1.26			0.01 ± 0.11			0.04 ± 0.20		
Anterior maxilla	50	103	0.71 ± 0.98			1.51 ± 1.08			0.03 ± 0.17			0.04 ± 0.18		
Type of augmentation														
None	3	8	1.29 ± 0.93	0.08	2.05	1.03 ± 0.87	0.5	0.82	2.11 ± 1.35	<0.001	32.52	1.86 ± 1.86	<0.01	2.10
Sinus lift	9	18	1.15 ± 1.21			1.09 ± 1.15			0.3 ± 0.51			0.07 ± 0.21		
Buccal augmentation for contour	54	251	0.68 ± 0.98			0.97 ± 1.13			0.15 ± 0.51			0.23 ± 0.68		
Massive augmentation	38	222	0.84 ± 0.98			1.11 ± 1.04			0.09 ± 0.31			0.08 ± 0.33		
Provisional restoration, healing cap, or cover screw														
Cover screw	40	115	0.62 ± 0.49	0.07	2.65	0.83 ± 1.1	0.06	2.76	0.43 ± 1.03	<0.001	16.36	0.55 ± 1.04	<0.001	24.79
Healing screw	4	18	1.12 ± 0.68			1.3 ± 0.76			0.01 ± 0.07			0.1 ± 0.24		
Provisional loading	51	367	0.81 ± 0.98			1.08 ± 1.09			0.09 ± 0.33			0.09 ± 0.4		
Time to definitive prosthesis														
<6 months	18	94	0.77 ± 1.05	0.67	0.38	1.03 ± 1.04	0.54	2.19	0.32 ± 1.35	<0.01	5.39	0.43 ± 0.98	<0.001	9.29
6 months-1 year	39	248	0.81 ± 0.99			1.13 ± 1.07			0.15 ± 0.46			0.18 ± 0.59		
>1 year	25	158	0.78 ± 0.96			0.87 ± 1.13			0.81 ± 0.29			0.08 ± 0.32		
Material for definitive prosthesis														
Emax restoration	19	43	0.70 ± 1.2	0.57	0.67	1.22 ± 1.34	0.14	1.81	0.43 ± 0.9	0.12	1.93	0.71 ± 1.04	<0.001	10.62
Full-contour zirconia	15	62	0.68 ± 0.85			0.77 ± 1.03			0.22 ± 0.49			0.21 ± 0.48		
Metal-ceramic	35	245	0.84 ± 0.9			1.09 ± 1.03			0.14 ± 0.62			0.15 ± 0.58		
BioHPP	21	149	0.74 ± 1.06			0.99 ± 1.11			0.11 ± 0.40			0.12 ± 0/53		
Type of prosthesis														
Splinted	4	8	0.31 ± 0.42	0.21	1.48	0.50 ± 0.62	0.21	1.49	0.26 ± 0.3	<0.001	9.63	0.11 ± 0.49	<0.001	17.25
Bridge	14	43	0.78 ± 0.94			0.79 ± 1.03			0.52 ± 1.32			0.52 ± 1.15		
Full-arch	65	397	0.81 ± 0.96			1.07 ± 1.07			0.09 ± 0.34			0.10 ± 0.43		
Single crown	22	51	0.57 ± 1.28			1.09 ± 1.3			0.32 ± 0.85			0.66 ± 0.97		

**Figure 3:**
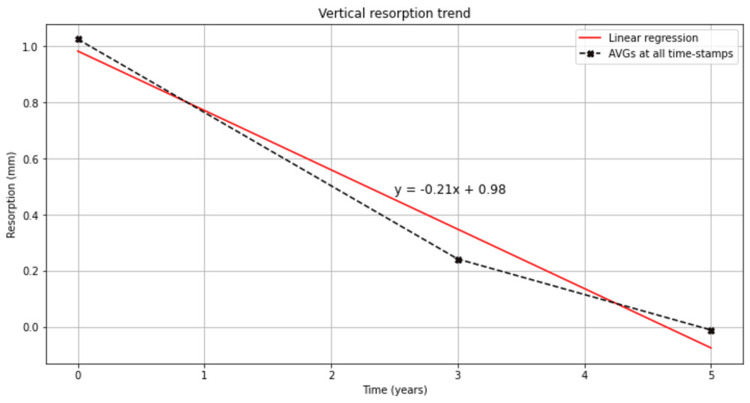
Bone resorption during the five-year follow-up

Eighteen patients who received 119 implants declared cigarette smoking habit, among which three patients declared the consumption of more than 20 cigarettes per day (heavy smokers). We did not observe any difference regarding the cigarette smoking and bone changes around the implants. Generally, the bone position after implant placement was stable at three and five years postoperatively (0.78 ± 0.99 mm at three years and 1.03 ± 1.09 mm at five years, Figure 3, Table 1). A correlation between bone resorption and age was seen; elderly patients above 60 years presented less bone remodeling compared with mature adults (35-60 years old). Also, a slight increase in bone resorption at five years was seen in female patients older than 50 years old (diagnosed at menopause) than the ones younger than 50 years old (0.85 ± 0.98 mm for women <50 years old vs 1.11 ± 1.49 mm for women >50 years old; p = 0.05).

**Figure 4:**
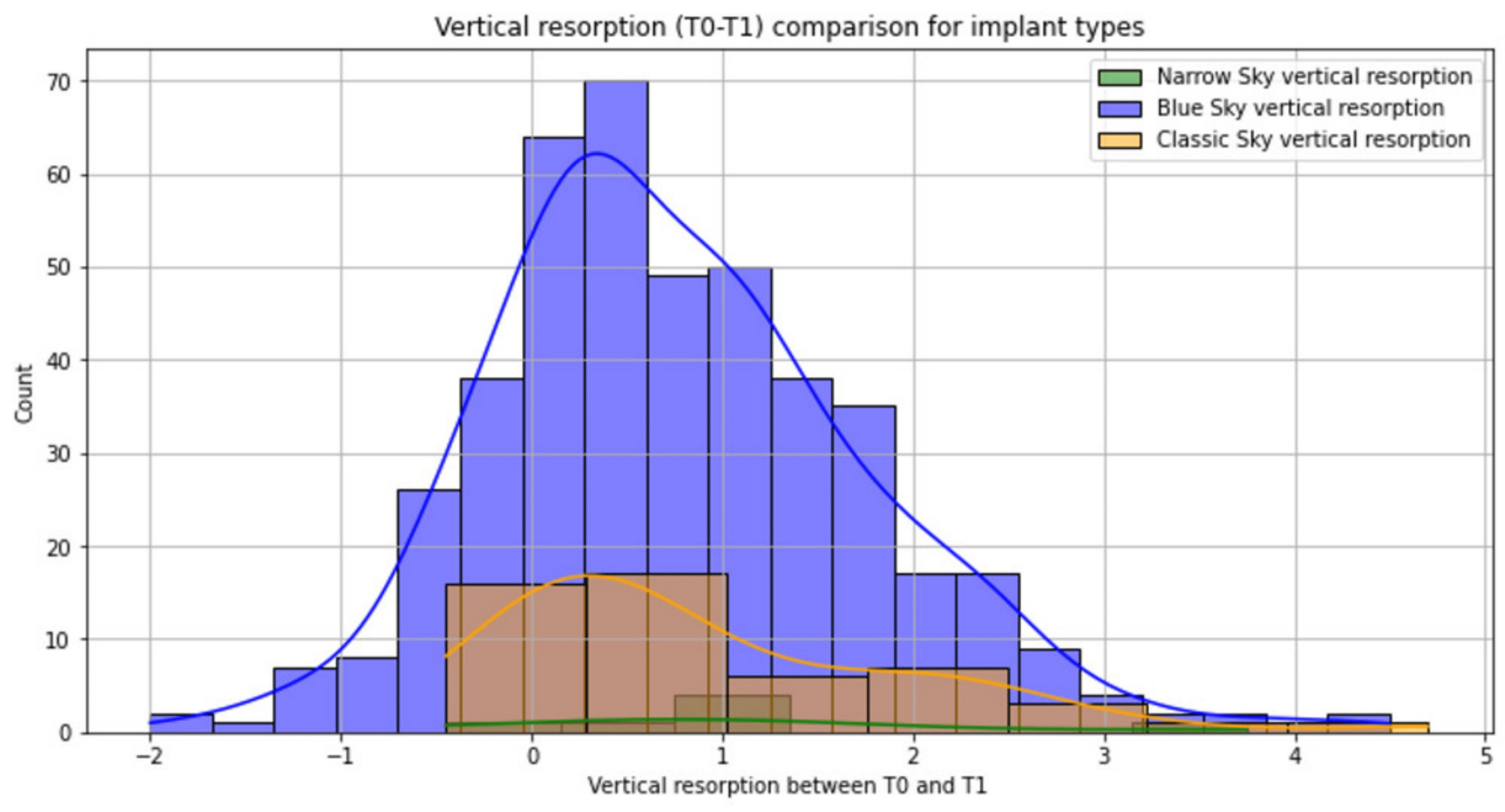
Bone remodeling on different types of implants at the three-year follow-up Blue Sky implants were the most used type of implants for rehabilitation with a wide distribution of bone resorption; Narrow Sky implants had the highest bone remodeling at three years postoperatively.

**Figure 5:**
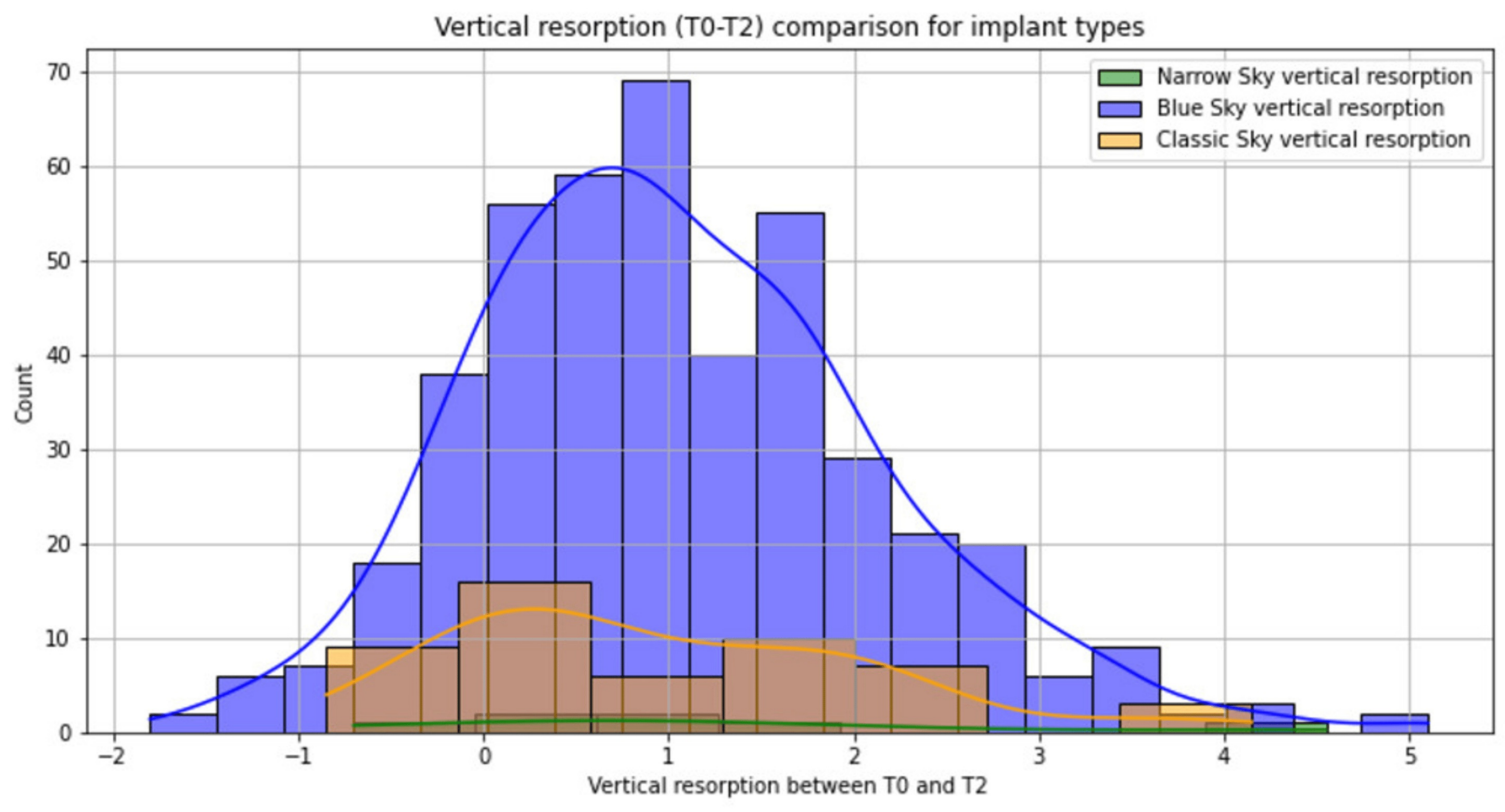
Bone remodeling on different types of implants at the five-year follow-up Blue Sky implants were the most used type of implants for rehabilitation with a wide distribution of bone resorption; Narrow Sky implants had the highest bone remodeling at five years postoperatively.

Blue Sky implants were the workhorse implants (Table 1). Blue Sky had a slightly reduced bone remodeling at three years (Figure 4), but with the same resorption as the other implant types at the five-year follow-up (Figure 5). No statistical significance was seen between the types of implants regarding bone resorption (p = 0.43). The 4-mm-diameter and 12-mm-long implants were the most frequent option. We observed statistically significant bone resorption for the 3.5-mm-diameter implant at three years (p = 0.008) and no further significant difference at the five-year follow-up (Table 1). Also, slightly reduced bone remodeling at three years was observed for 4.5-mm-diameter implants and 8-mm-long implants, but with no statistical significance (p > 0.05). Most of the implants were inserted 1 mm under the crestal bone, which had the lowest bone resorption compared with more than 1 mm depth of implant placement.

Maxillary implants had lower bone resorption compared to mandible implants (p = 0.09) for both the three- and five-year follow-ups. Moreover, the anterior mandible had the highest bone remodeling at the three- and five-year follow-ups, compared to other anatomical regions (p < 0.001; Table 1).

Except for three patients with eight implants, all the patients needed various amounts of bone augmentation. These three patients had the highest bone remodeling process at the three-year follow-up. Also, patients who required large bone augmentation had higher values of marginal bone loss compared to the others. For example, sinus lifting augmentations and large lateral bone augmentations had increased bone resorptions at three years (1.15 ± 1.21 mm and 0.84 ± 0.98 mm) and five years (1.09 ± 1.15 mm and 1.11 ± 1.04 mm) compared to minimal grafting for buccal contour (0.68 ± 0.98 mm at three years and 0.97 ± 1.13 mm at five years, Table 1). However, no statistical difference was seen.

The patients who received gingiva healing screws or provisional prosthetics had increased bone remodeling compared to the ones with cover screws (Table 1). Commonly, patients who had provisional restorations were patients with full-arch rehabilitation or the ones with implants applied in the aesthetic area. Most patients received the final restorations between six months and one year after the implant placement. Almost similar bone resorptions were seen regardless of the time to definitive prosthesis. Splinted prostheses using MUA had lower bone resorptions (0.31 ± 0.42 mm) compared to full-arch rehabilitation (0.81 ± 0.96 mm; p = 0.21). Also, full-contour zirconia had the lowest bone remodeling at three years (0.68 ± 0.85 mm) and five years (0.77 ± 1.03 mm) compared to other materials (three years = 0.79 ± 1.01 mm and five years = 1.07 ± 1.09 mm; p = 0.04).

We also evaluated the horizontal resorption around the implants (Table 1). We observed that the tissue-level implants (Classic Sky) had statistically significant horizontal bone remodeling compared to other implants (Table 1). Posterior mandible, no bone augmentation, cover screw, and bridge final prosthesis were the other factors that may statistically increase the horizontal bone resorption.

## Discussion

This study succeeded in evaluating the implant- and patient-specific factors that contribute to the bone remodeling around the Bredent implants. The accepted bone resorption around implants is 1 mm in the first year and 0.2 mm furthermore [6]. The mean bone resorption per implant in our study was approximately 1 mm at the five-year follow-up, with approximately 0.78 mm of them developing in the first three years. The workhorse of the implant rehabilitation was the bone-level implant Blue Sky. Only slightly reduced bone resorption was seen in this type of implant compared to the others. The literature is heterogeneous regarding the difference in marginal bone loss around the implants between bone-level and tissue-level implants, with the platform-switching implant being the paradigm shift of implant-specific factors that may better conserve the bone [28]. Another key factor that may influence bone resorption is the implant dimension, considering that a large bone-to-implant connection can provide better implant stability [22]. In our study, we observed that the narrowest diameter (3.5 mm) produced increased resorption in comparison with the other implants. Unexpectedly, minimal resorption was seen in the 8-mm-long implants compared with longer implants. However, the dimension of the implants has to be wisely chosen keeping in mind various factors, including bone dimension, bone augmentation, 3D implant position, implant angulation, etc. [22]. We recommend using at least a 4-mm-diameter implant in any suitable situation.

The protocol of insertion and the treatment planning, from choosing the implant type to the prosthesis material, are very important for the success of implant-based therapy. We respected the drilling protocol of the Bredent manufacturer, but the depth of insertion was dependent on local bone factors, such as bone geometry, bone availability, the position and angulation of the implant, or adjacent anatomical structures (e.g., mandibular canal or maxillary sinus) [15,16]. We observed that the insertion depth of approximately 1 cm had the best results regarding bone remodeling stability. Usually, in the post-extraction sites, the implant insertion is deeper due to the higher expected bone resorption. Therefore, the increased value of bone remodeling in a 1-2 mm depth of insertion can be explained by this intense remodeling process. Also, marginal bone loss around implants can be different in the maxilla and mandible due to various factors, such as different bone densities or physiological patterns of resorption [17]. We observed that the mandible has higher bone resorption in comparison with the maxilla, with the anterior mandible having the most increased bone resorption. On the one hand, this finding is contrary to the general knowledge which indicates that a more corticated bone (e.g., mandible) has less remodeling [17,18]. On the other hand, the increased resorption can be explained by a higher surgical bone preparation during implant insertion, especially in an All-on-X concept in post-extraction sites, where the bone level has to be similar between implants and the implants are inserted in deeper positions [19-21]. However, we recommend a deeper implant insertion in the anterior mandible in any suitable situation. Moreover, the bone augmentation of the implant site is a critical factor for bone remodeling [22,23]. In our study, we observed that the highest bone resorption was seen in the non-augmented sites, in both vertical and horizontal directions, and less resorption was found in the buccal augmentation for bony contour.

Prosthodontic factors are also important when discussing marginal bone loss around implants [29,30]. Starting with the decision of immediate loading, healing screw, or cover screw, the bone will have different types of resorptions [31]. For example, we observed that the lowest vertical bone remodeling was seen in the cover screw option, but with the disadvantage of the highest horizontal resorption. Conversely, at the five-year follow-up, there was a minimal difference between vertical resorption in immediately loaded implants and cover screws. We also observed no significant difference in time to the definitive prosthesis, similar to other papers [32]. Prosthetic materials and prosthodontic strategy may also interfere with bone resorption. We observed reduced bone resorption around full-contour zirconia restorations compared with other types of materials, with these results being different from the findings of the literature [33,34]. However, the success may depend on the manufacturer's options and prosthetic design [35]. We observed that splinted implants provide the most stable bone around the implant during the time compared to other variants, with the results from the literature being heterogeneous [36,37]. However, bone remodeling depends on various factors, such as the type of prosthesis retention (cemented vs screw-retained prosthesis) [37]. We used only screw-retained prostheses. Hence, we recommend screw-retained zirconia splinted prosthesis in any suitable situation.

We analyzed also age, sex, and smoking as patient-related factors that may influence bone resorption. We did not see differences between male and female patients, as well as cigarette-smoking and non-cigarette-smoking patients, with the results being contradictory to the literature, including other Romanian cohorts [33]. It is generally accepted that cigarette smoking induces higher bone resorption around implants and may produce implant failure [8,9]. However, a larger cohort and a more balanced ratio between cigarette-smoking and non-cigarette-smoking patients can have different results. Age is related to bone metabolism and bone remodeling around the implants. Older patients have an increased bone deficiency metabolism, which leads to an increase in bone resorption [5,33]. Contrary to this literature knowledge, we observed that patients younger than 60 years old have higher levels of bone remodeling near the implants compared with older patients. Also, female patients above 50 years old are prone to have more bone remodeling than the ones younger than that. This may be explained by the bone metabolism swift that appears during the menopause period [5].

Study particularities and limitations

We included only one implant system (Bredent Medical GmbH & Co., Senden, Germany) due to the fact that different systems could induce various types of bone resorptions. Therefore, the research could focus on specific patterns of bone remodeling related to the bone-implant connections excluding brand-specific changes [24]. Also, the surface treatment and overall production process of one system are similar, regardless of the type of implant (bone level or tissue level). This is a radiological-limited research related to implant marginal bone loss. Other research combined radiological and clinical evaluation to add the investigation of implant-related complications, such as peri-implantitis, to the evaluation of bone resorption near the implants [7,38]. In our study, we used OPG instead of CBCT or retro-alveolar radiographs for follow-up. This was due to the standard follow-up protocol that minimizes the radiation dose, being in accordance with other research [27,39]. However, we were aware of the CBCT advantages, and even if OPG is a 2D investigation that can have some disadvantages (e.g., image distortion), we used the same radiological system (OPG) and the same examination protocol that could offer interobserver reliability and precision of measurement in different periods. Also, implant stability quotient (ISQ) and Periotest instruments were not used for implant evaluation. Another limitation of this study is the lack of investigation of bone remodeling related to factors such as the type of bone (native bone or post-extraction bone sites) or various anatomical shapes of bone, as well as the soft tissue types adjacent to the implants. However, the aim of our study was to identify implant- and patient-specific factors that influence bone resorption. There is a need for more research to fully understand this multifactorial process. The last particularity is the inclusion of patients in general good health to overcome the bone changes associated with various systemic conditions, similar to other papers [11,26].

## Conclusions

Bone remodeling around implants is a multifactorial process influenced by many implant- and patient-specific factors. Narrow implants induce increased bone resorption, as well as the 3D implant positioning in the anterior mandible. Contrarily, implant depth of insertion of approximately 1 mm, splinted implants, and zirconia materials can reduce bone remodeling. Future research that includes long-term analysis and various implant systems is required but with the perspective of using the same protocol of implant insertion and follow-up.
